# Molecular mechanisms of lncRNAs in regulating cancer cell radiosensitivity

**DOI:** 10.1042/BSR20190590

**Published:** 2019-08-28

**Authors:** Jiamin Zhu, Shusen Chen, Baixia Yang, Weidong Mao, Xi Yang, Jing Cai

**Affiliations:** 1 Department of Oncology, the Affiliated Jiangyin Hospital of Southeast University Medical College, 163 Shoushan Road, Jiangyin 214400, P.R. China; 2 Department of Radiation Oncology, Nantong Tumor Hospital, Affiliated Tumor Hospital of Nantong University, Nantong 226321, China; 3 Department of Radiation Oncology, Fudan University Shanghai Cancer Center, Department of Oncology, Shanghai Medical College, Fudan University, Shanghai 200032, China

**Keywords:** apoptosis, cancer, epithelial-mesenchymal transition, lncRNA, radioresistance, radiotherapy

## Abstract

Radiotherapy is one of the main modalities of cancer treatment. However, tumor recurrence following radiotherapy occurs in many cancer patients. A key to solving this problem is the optimization of radiosensitivity. In recent years, long non-coding RNAs (lncRNAs), which affect the occurrence and development of tumors through a variety of mechanisms, have become a popular research topic. LncRNAs have been found to influence radiosensitivity by regulating various mechanisms, including DNA damage repair, cell cycle arrest, apoptosis, cancer stem cells regulation, epithelial–mesenchymal transition, and autophagy. LncRNAs are expected to become a potential therapeutic target for radiotherapy in the future. This article reviews recent advances in the role and mechanism of lncRNAs in tumor radiosensitivity.

Among various RNA species, non-coding RNAs are RNA molecules that transcribe but do not encode proteins. LncRNAs are a group of RNAs, ranging in length from 200 to 100,000 nucleotides, which participate in diverse cellular processes and are involved in disease progression [1]. LncRNAs are often classified into five categories: antisense lncRNA, intronic transcript lncRNA, large intergenic non-coding RNA (lincRNA), promoter-associated lncRNA, and untranslated region-associated lncRNA [2]. LncRNAs are mainly localized to the cell nucleus or cytoplasm [3]. Nuclear lncRNAs can regulate the chromatin architecture of cells by interacting with chromatin remodeling complexes. They also regulate gene expression on the same chromosome [4]. The most common mechanism cytoplasmic lncRNAs use to regulate genes is to act as a competitive endogenous RNA (ceRNA). CeRNAs are reported to function as microRNA (miRNA) sponges by binding specific complementary sequences and protecting target miRNA from being repressed by miRNA [5]. LncRNAs affect biological pathways in various cancers through different mechanisms and influence the expression of target genes at both the transcriptional and post-transcriptional levels.

Radiotherapy is an effective treatment method for many cancers, and approximately half to two-thirds of all patients with cancer receive this type of therapy [6,7]. However, radioresistance is a primary factor that leads to poor prognosis. Sensitivity to radiotherapy is the key to its therapeutic effect on malignant tumors and is a complex process associated with multiple genes, factors, and mechanisms. During radiotherapy, ionizing radiation first induces water radiolysis to produce reactive oxygen species (ROS). Oxygen then provides unpaired electrons for free radicals in DNA molecules, thereby stabilizing infrared-induced DNA damage. Damaged DNA or excessive ROS activate apoptotic signaling pathways in cancer cells, leading to cell death [8]. The effect of radiotherapy is determined by following the 4’Rs of radiobiology: repair of DNA damage, redistribution of the cell cycle, repopulation of tumors, and reoxygenation of hypoxic tumor areas [9]. Radioresistance can be overcome by reducing DNA repair and decreasing DNA damage tolerance through the activation of intracellular pro-survival and antiapoptotic signaling pathways. Previous studies have indicated that lncRNAs influence radioresistance through mechanisms (Table 1 and Figure 1) that include repair of DNA damage, cell cycle arrest, apoptosis, CSCs regulation, epithelial–mesenchymal transition (EMT), and autophagy. In this review, we summarize research on several representative mechanisms of lncRNAs in radiation therapy.

**Figure 1:**
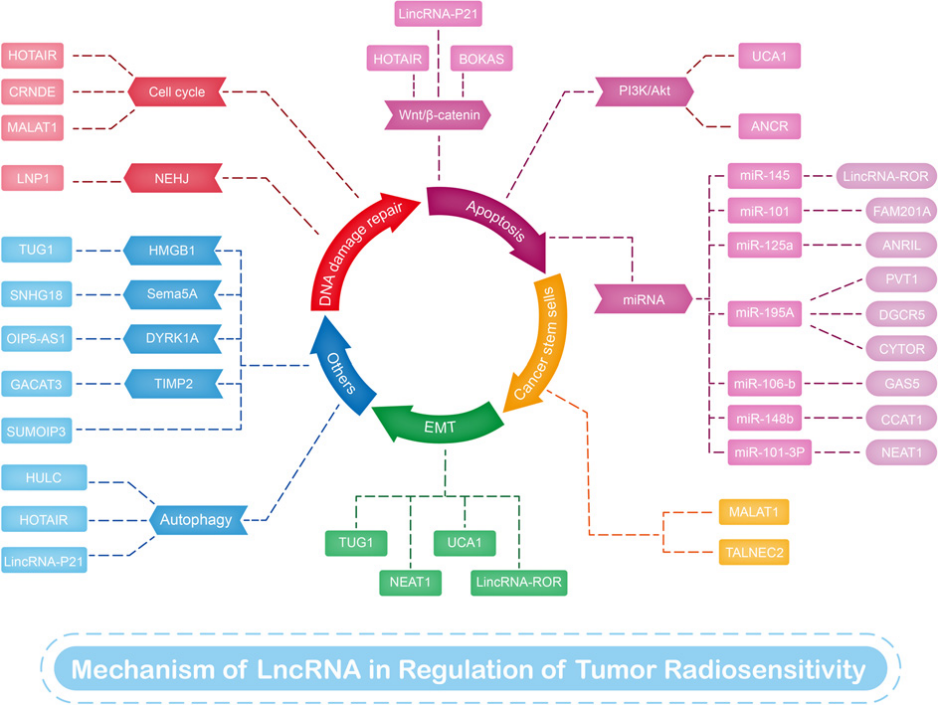
LncRNAs involved in radiosensitivity of various cancers This figure summarizes the mechanisms and targets of lncRNAs in regulating radiosensitivity of radiotherapy.

**Table 1:** Summary of lncRNAs that regulate radiosensitivity in radiation therapy

LncRNA	Radiosensitivity	Types of tumor	Mechanism	Molecular target	Reference
LINP1	Down	Cervical cancer	DNA damage repair	N/A	[13]
MALAT1	Down	Cervical cancer	DNA damage repair	miR-145	[15]
	Down	ESCC	DNA damage repair	CKS1	[16]
	Down	NPC	CSC	miR-1/slug	[54]
HOTAIR	Down	Cervical caner	DNA damage repair	p21 protein	[19]
	Down	Pancreatic ductal cancer	Wnt/β-catenin pathway	WIF-1	[25]
	Down	Pancreatic cancer	Autophagy	Atg7	[69]
CRNDE	Down	Lung cancer	DNA damage repair	p21 protein	[20]
BOKAS	Down	ESCC	Wnt/β-catenin pathway	WISP1	[28]
LincRNA-p21	Up	Colorectal cancer	Wnt/β-catenin pathway	N/A	[31]
	Up	Gastric cancer	Wnt/β-catenin pathway	N/A	[32]
	Down	Hepatoma and glioma cancer	Autophagy	N/A	[71]
UCA1	Down	Prostate cancer	P13k/Akt pathway	N/A	[35]
ANCR	Down	NPC	P13k/Akt pathway	N/A	[36]
LincRNA-ROR	Down	Colorectal cancer	Apoptosis	p53/miR-145	[39]
ANRIL	Down	NPC	Apoptosis	miR-125a	[40]
CCAT1	Down	Breast cancer	Apoptosis	miR-148b	[41]
OIP5-AS1	Up	Colorectal cancer	Apoptosis	DYRK1Y/miR-369-3p	[43]
PVT1	Down	Lung cancer	Apoptosis	miR-195	[44]
DGCR5	Down	Laryngeal cancer	Apoptosis	miR-195	[45]
CYTOR	Down	Lung cancer	Apoptosis	miR-195	[46]
NEAT1	UP	NPC	Apoptosis	miR-101-3b	[47]
	Down	NPC	EMT	miR-204/ZEB1	[61]
GAS5	Up	Cervical cancer	Apoptosis	miR-106b	[48]
TALNEC2	Down	Glioma	CSC	N/A	[56]
TUG1	Down	Bladder cancer	EMT	miR-145/ZEB2	[60]
	Down	Bladder cancer	N/A	HMGB	[74]
HULC	Down	Prostate cancer	Autophagy	N/A	[71]
SNHG18	Down	Glioma	N/A	Semaphorin 5A	[73]
SUMO1P3	Down	Hepatocellular cancer	N/A	N/A	[75]
GACAT3	Up	Lung cancer	N/A	TIMP2	[76]

N/A: The corresponding mechanisms and targets are not yet clear, and need further exploration.

## DNA damage repair

Ionizing radiation can generate high ROS known as free radicals and cause DNA double-strand breaks that ultimately lead to extensive genomic instability and cell death. After DNA damage, cells initiate a repair system to fix DNA damage, in turn reducing sensitivity to radiotherapy [10]. DNA damage can be repaired either by homologous recombination or through non-homologous end joining (NHEJ). The latter is a major pathway for the repair of damaged DNA and is a key determinant of infrared (IR) resistance in cancer cells [11]. Zhang et al. [12] found that lncRNA in the NHEJ pathway 1 (LINP1) accelerated NHEJ repair and decreased the sensitivity of tumors to ionizing radiation by staining for γ-H2AX to assess the level of double-strand breaks. Wang et al*.* [13] also revealed that in cervical cancer, LINP1 enhanced the efficiency of DNA damage repair via the NHEJ pathway and decreased radiosensitivity.

Following IR-induced DNA damage, molecules at cell cycle checkpoints begin to regulate and arrest cell cycle progression, subsequently repairing damaged DNA or initiating apoptosis if this repair is unsuccessful. Cell cycle arrest is closely related to radiosensitivity, and when cancer cells are arrested in the G2/M phase, their radiosensitivity increases [14]. More researchers have found that lncRNAs regulate radiosensitivity by affecting DNA damage repair via cell cycle arrest. Lu et al*.* [15] found that knockdown of metastasis-associated lung adenocarcinoma transcript 1 (MALAT1) negatively regulated miR-145 levels and increased radiosensitivity. In another study, the authors reported that the downregulation of MALAT1 affected DNA repair by inducing G2/M arrest, which is vital in radiotherapy. Moreover, Li et al*.* [16] showed that MALAT1 induced the radioresistance of esophageal squamous cell carcinoma (ESCC) cells by down-regulating the expression of cdc kinase subunit 1 (CKS1), which is a regulatory protein that induces G2/M arrest by inhibiting p27 [17]. Other studies showed that p53/p21 activation induced G1 arrest [18] and that the suppression of p53 activation promoted G2/M phase arrest. Jing et al. [19] demonstrated that hox transcript antisense intergenic lncRNA (HOTAIR) increased radioresistance by inhibiting the expression of p21 and inducing cell cycle arrest at the S phase. Likewise, Zhang et al. [20] found that the colorectal neoplasia differentially expressed (CRNDE) gene contributed to radioresistance in lung cancer cells by suppressing p21 expression and affecting the G1/S transition. The aforementioned studies illustrated that lncRNAs could regulate the radiosensitivity of various cancers such as cervical cancer, ESCC, and lung cancer by affecting DNA repair via accelerating NHEJ repair, regulating the expression of p53/p21 and affecting cell cycle arrest. However, the mechanism underlying lncRNA-mediated regulation of DNA damage repair is complicated and the related studies have only been conducted *in vitro*. More *in vivo* models therefore need to be established to further study these mechanisms.

## Apoptosis

Apoptosis, also known as programmed cell death, is the main mode of cell death after irradiation [21], and is regulated by both intrinsic and extrinsic pathways. The intrinsic pathway responds to signals such as ultraviolet radiation or DNA damage and activates ‘executioner’ caspases through a mitochondria-dependent pathway [22]. Radiation can trigger apoptosis intrinsically or extrinsically by causing DNA damage or other types of severe cellular injury such as reactive oxidative stress, thus leading to downstream gene cascade reactions involving Bcl-2, caspases, Wnt/β-catenin, and other molecular signals [23]. The Wnt/β-catenin signaling pathway is a pro-survival pathway that facilitates extensive crosstalk with other signaling pathways such as phosphatidylinositol 3-kinase/protein kinase B (PI3K/Akt) and signal transducers and activators of transcription (STAT). Accumulating evidence indicates that the Wnt/β-catenin pathway regulates radiosensitivity by participating in proliferation and apoptosis [24]. Jiang et al*.* [25] found that HOTAIR affected the radiosensitivity of pancreatic ductal adenocarcinoma cells via Wnt inhibitory factor 1, which was demonstrated to be a primary inhibitor of the Wnt/β-catenin pathway [26]. In lung cancer radiotherapy, radiosensitivity can be reduced by inactivating β-catenin, mediated by HOTAIR upregulation [27]. The gene encoding Wnt1-inducible signaling pathway protein 1 (WISP1) is expressed in response to Wnt1 and β-catenin and mediates radioresistance both *in vitro* and in xenograft tumor models. LncRNA BOKAS was found as a natural antisense transcript of BOK, Zhang et al*.* [28] found that BOKAS promoted WISP1 upregulation and induced radioresistance. Moreover, lincRNA-p21, a downstream agent of p53 [29], downregulated the expression of β-catenin at the post-transcriptional level [30], and increased the sensitivity of colorectal cancer [31] and gastric cancer cells [32] to radiotherapy via the Wnt/β-catenin signaling pathway.

PI3K/Akt is another critical signal pathway influencing cellular proliferation, apoptosis, and progression [33]. Its activity induces the resistance of human cancer cells to radiation therapy via three main mechanisms: intrinsic radioresistance, tumor cell proliferation, and hypoxia [34]. Ghiam et al*.* [35] observed that knockdown of urothelial carcinoma associated 1 (UCA1) in prostate cancer cells could induce radiosensitivity by reducing Akt activation. Recently, Ma et al*.* [37] eported that anti-differentiation non-coding RNA (ANCR) promoted radioresistance by inhibiting phosphatase and tensin homolog (PTEN) [36], which was confirmed to regulate the PI3K/Akt pathway.

The majority of miRNAs play vital roles in regulating biological processes such as cell differentiation and apoptosis [38]. In recent studies, lncRNAs were reported to function as miRNA sponges to regulate radiosensitivity in cancer cells. Yang et al. [39] discovered that lincRNA–ROR reduced the radiosensitivity of colorectal cancer cells by suppressing cell viability and promoting apoptosis via activation of p53/miR-145. Similarly, antisense non-coding RNA in the INK4 locus (ANRIL) enhanced the radioresistance of nasopharyngeal carcinoma (NPC) cells by targeting miR-125a [40]. LncRNA colon-cancer-associated transcript-1 (CCAT1) also improved the radioresistance of breast cancer cells by negatively regulating miR-148b expression [41]. Family with sequence similarity 201-member A (FAM201A) was reported to mediate the radiosensitivity of ESCC by regulating ataxia telangiectasia mutation and mammalian target of rapamycin (mTOR) expression via miR-101 [42]. The dual-specificity tyrosine phosphorylation-regulated kinase-1A (DYRK1A) functions as both a tumor-suppressing and oncogenic factor in various cancers. LncRNA opa-interacting protein 5 antisense RNA 1 (OIP5-AS1) was found to increase the radiosensitivity of colorectal cancer cells by regulating the expression of DYRK1A and miR-369-3p [43]. Coincidentally, the lncRNAs plasmacytoma variant translocation 1 (PVT1), diGeorge syndrome critical region gene 5 (DGCR5), and cytoskeleton regulator RNA (CYTOR) were confirmed to decrease the radiosensitivity of non-small cell lung cancer cells by sponging miR-195 [44–46]. On the other hand, other lncRNAs increase radiosensitivity via miRNA targeting. For instance, Wang et al. [47] found that nuclear enriched abundant transcript 1 (NEAT1) enhanced the radiosensitivity of NPC cells and promoted their apoptosis by targeting miR101-3p. Moreover, growth arrest special 5 (GAS5) increased the radiosensitivity of cervical cancer cells by inhibiting miR-106b [48]. Collectively, these findings indicated that lncRNAs could affect the proliferation and apoptosis of cancer cells by regulating related signaling pathways, such as the Wnt/β-catenin pathway and PI3K/Akt pathway, or by acting as miRNAs sponges, thereby affecting the radiosensitivity of cancer cells. The discovery of more signaling pathways and miRNAs involved in this mechanism is needed to potentially improve the radiosensitivity of cancer cells.

## CSCs

CSCs are small subpopulations of cancer cells that can renew themselves and have the ability to generate heterogeneous cell lineages that make up the tumor [49]. During malignant transformation, CSCs display specific molecular characteristics and properties, which may be related to tumor invasiveness, resistance to treatment, and the tendency toward metastasis and diffusion [50]. CSCs are strongly related to tumor aggressiveness and treatment response, and eliminating CSCs can cure cancer [51]. CSCs are reported to promote radioresistance via various pathways that include activating the DNA damage response, scavenging intracellular ROS, inducing hypoxia, and modulating the microenvironment. Increasing evidence indicated that CSCs contributed to radioresistance, which was associated with both intrinsic and extrinsic determinants and could result in the failure of radiation treatment [52]. The intrinsic determinants reportedly include ROS levels, DNA repair capability, cell cycle status, apoptosis, autophagy, and regulation of survival pathways, whereas extrinsic determinants involve hypoxic microenvironments [53]. Thus, CSCs have been investigated in basic cancer research and these studies are rapidly expanding into many related aspects including radiosensitization. The results of recent investigations suggested the possibility that lncRNAs may influence cancer radiotherapy by regulating CSCs. Jin et al*.* [54] examined the radiosensitivity of NPC cells and demonstrated that MALAT1 expression was increased in NPC cell lines. They observed that MALAT1 decreased the sensitivity of NPC cells to IR by modulating CSC activity and regulating the miR-1/slug axis. Glioma stem cells (GSCs) are a small subpopulation of CSCs that have been shown to be involved in tumor infiltration, resistance to cancer therapy, and tumor recurrence. Previous investigations have shown that CSCs promote glioma radioresistance via preferential activation of the DNA damage checkpoint response and upregulation of DNA repair capacity [55]. Recently, Brodie et al*.* [56] demonstrated that tumor-associated long non-coding RNA expressed on chromosome 2 (TALNEC2) promoted the tumorigenic potential and mesenchymal transformation of GSCs and increased their resistance to radiation. All of the above-mentioned findings showed that multiple mechanisms contributed to the role of CSCs in radioresistance. LncRNAs such as MALAT1 and TALNEC2 can increase the radiation resistance of NPC cell lines and glioma cells by regulating the activity of CSCs. However, very few studies on the effect of lncRNAs on cancer radiotherapy through CSCs have been conducted, and these models are still in the experimental stages *in vitro*. A better understanding of CSCs and associated lncRNAs that regulate radioresistance could potentially lead to new treatment strategies for cancer radiotherapy.

## EMT

EMT is an important biological process through which epithelial cells lose their polarity and are converted into the mesenchymal phenotype. EMT is an embryonic procedure that induces the loss of cell–cell contact and invasion, and is associated with resistance to chemotherapeutic drugs and radiation [57]. Cells undergoing EMT acquire mesenchymal traits with the upregulation of N-cadherin, vimentin, and transcription factors including snail, slug, zinc finger E-box-binding homeobox 1 (ZEB1), and zinc finger E-box-binding homeobox 2 (ZEB2) [58]. Snail and slug inhibit p53-mediated apoptosis in response to IR, whereas ZEB1 promotes IR-induced DNA damage repair. EMT contributes to radioresistance by inducing hypoxia, increasing DNA repair ability and activating growth factor pathways [59]. LncRNA taurine up-regulated gene 1 (TUG1) has been shown to induce radioresistance by promoting EMT and targeting the miR-145/ZEB2 axis [60]. Likewise, Lu et al*.* [61] reported that NEAT1 knockdown reversed the EMT phenotype by targeting miR-204/ZEB1 in NPC cells, indicating that NEAT1 serves as an EMT inducer and can lead to the radioresistance of NPC cells. Yang et al*.* [62] demonstrated that downregulating UCA1 reduced the expression of EMT markers such as matrix metalloproteinase (MMP)-2, MMP-9, ZEB1, and vimentin. The authors concluded that the downregulation of UCA1 could induce radiosensitivity in colorectal cancer cells by suppressing EMT. Another study showed that lincRNA–ROR induced EMT [63], but whether it promoted radioresistance by regulating EMT has not been studied. As mentioned above, lncRNAs can regulate the radiosensitivity of cancer cells by inducing or inhibiting EMT through different mechanisms. This suggests that EMT is involved in radioresistance and that specifically targeting EMT can provide a novel solution to improve the therapeutic effectiveness of radiation against cancer.

## Other mechanisms

Autophagy has been identified as a key catabolic process that contributes to the maintenance of cellular homeostasis via the decay of damaged or unwanted proteins and dysfunctional cytoplasmic organelles [64]. Autophagy is a procedure of cellular self-consumption that begins with Unc-51-like kinase (ULK) activity in the ULK/ATG13/ATG101/FIP-200 (RB1CC1) complex. At various stages, autophagy is regulated by multiple pathways such as PI3K/Akt/mTOR complex 1 and AMP-activated protein kinase. Autophagy has two opposite functions associated with radiation stress in cancer cells. One of these is cytoprotection, inhibition of which renders cancer cells sensitive to treatment, and the other is cytotoxicity, which promotes cancer cell death [65]. Several investigations have demonstrated that inhibiting autophagy in cancer cells, such as in ESCC and non-small cell lung cancer (NSCLC), contributes to the radiosensitivity of tumor cells [66,67]. Autophagy is reported to contribute to radioresistance through factors such as the degree of tumor hypoxia, the presence of CSCs, and the ability to repair DNA damage [68]. LncRNAs reportedly regulate cancer cell radiosensitivity by modulating different autophagic effects. HOTAIR, mentioned in the previous section, was found to decrease radiosensitivity by promoting autophagy via the upregulation of Atg7 expression in pancreatic cancer cells [69]. Moreover, Chen et al*.* [70] demonstrated that lncRNA highly upregulated in liver cancer (HULC) reduced autophagy by interacting with Beclin-1 and inhibiting mTOR, thereby decreasing the sensitivity of prostate cancer cells to irradiation. In addition, Shen et al*.* [71] observed that lincRNA-p21 decreased radiosensitivity in hypoxic hepatoma and glioma cells by downregulating autophagy through the hypoxia-inducible factor-1/Akt/mTOR/P70S6K pathway in hypoxic tumor cells. Collectively, these data show the complex crosstalk between autophagy and lncRNAs that is relevant in radiotherapy. However, more complex experimental studies are required to further elucidate the various mechanisms.

Other lncRNAs have been reported to regulate radiosensitivity by targeting specific molecules that are claimed to be involved in radiotherapy. Small nucleolar RNA host gene 18 (SNHG18) is significantly upregulated in clinical glioma tissues and is negatively associated with semaphorin 5A (Sema5A) expression. A recent study showed that inhibiting SNHG18 reduced the radioresistance of glioma cells via Sema5A [72]. Another study found that knockdown of high mobility group box 1 (HMGB1) led to greater DNA damage and enhanced radiosensitivity [73]. LncRNA TUG1 knockdown enhanced the radiosensitivity of bladder cancer cells by reducing the expression of HMGB1 [74]. Zhou et al*.* reported that small ubiquitin-like modifier 1 pseudogene 3 (SUMO1P3) reduced radiosensitivity in hepatocellular carcinoma cells [75], whereas the lncRNA gastric cancer-associated transcript 3 (GACAT3) enhanced radiosensitivity by targeting tissue inhibitor of metalloproteinases 2 (TIMP2) [76]. However, the mechanisms of these lncRNAs need to be further investigated to identify their specific association with radiotherapy.

## Discussion and perspectives

Radiotherapy is an important cancer treatment, but its effectiveness is limited by radioresistance. The underlying mechanisms behind radioresistance have not been fully elucidated. Overcoming radioresistance is one of the main challenges in cancer research. This review has summarized the lncRNAs that are associated with radiosensitivity via phenomena that include DNA damage repair, apoptosis, CSC regulation, EMT and autophagy.

LncRNAs have been demonstrated to be involved in a variety of mechanisms, such as transcriptional regulation, protein modification, translation and formation of RNA-protein or protein-protein complexes. Many researchers believe that lncRNAs alone might not be sufficient to drive cell signaling, and that in turn, cell signaling might not be triggered without lncRNAs [77]. Studies on lncRNAs are of great significance in exploring the molecular signaling underlying complex radioresistant processes. Recently, advances of lncRNAs expressions in cancers have highlighted the potential roles as biomarkers in diagnosis and prognosis of the patients [78,79]. At present, there has been no study on the relationship between radiotherapy efficacy and lncRNAs as biomarkers in radiotherapy patients. Therefore, investigations of clinical radiotherapy sensitivity and lncRNAs are necessary to directly prove the relationship between lncRNAs and radiotherapy sensitivity of cancer cells. In addition, in clinical application, lncRNAs have also been recognized as oncogenes or tumor suppressor genes that can regulate the sensitivity of cancer cells to anticancer regimens [80]. LncRNAs present a potential molecular biomarker in radiotherapy and targeted molecular agents against lncRNAs would be a promising new way to treat radioresistance of cancers from current, as evidenced by both *in vitro* and *in vivo* studies. However, more basic and clinical studies are needed to investigate how lncRNAs and signaling molecules work together to influence various aspects of radiosensitivity as well as to study the application value of lncRNAs in radiotherapy.

## Abbreviations

ANCR: anti-differentiation non-coding RNA
ANRIL: antisense non-coding RNA in the INK4 locus
CCAT1: colon-cancer-associated transcript-1
CRNDE: colorectal neoplasia differentially expressed
DGCR5: DiGeorge syndrome critical region gene 5
HOTAIR: hox transcript antisense intergenic lncRNA
MALAT1: metastasis-associated lung adenocarcinoma transcript 1
NEAT1: nuclear enriched abundant transcript 1
NHEJ: non-homologous end joining
PTEN: phosphatase and tensin homolog
PVT1: plasmacytoma variant translocation 1
TIMP2: tissue inhibitor of metalloproteinases 2
UCA1: urothelial carcinoma associated 1
WISP1: Wnt1 inducible signaling pathway protein 1
